# AEROBIC TRAINING, THE DEFAULT MODE NETWORK, AND COGNITION IN OLDER ADULTS WITH MILD VASCULAR COGNITIVE IMPAIRMENT

**DOI:** 10.1093/geroni/igz038.213

**Published:** 2019-11-08

**Authors:** Rachel A Crockett, Chun Liang Hsu, Cindy Barha, Ging-Yuek Robin Hsiung, Teresa Liu-Ambrose

**Affiliations:** University of British Columbia, Vancouver, British Columbia, Canada

## Abstract

Aerobic training has been shown to be effective at improving cognitive and brain outcomes in older adults with mild subcortical ischemic vascular cognitive impairment (SIVCI). However, uncertainty remains regarding the underlying neurobiological mechanisms by which exercise elicits these improvements in cognition. Increased aberrant functional connectivity of the default mode network has been highlighted as a factor contributing to cognitive decline in older adults with cognitive impairment. Greater connectivity of the DMN at rest is associated with poorer performance on attention-demanding tasks, indicative of a lack of ability to deactivate the network on task. Our previous work on a randomized controlled trial of participants with mild SIVCI, demonstrated that 6-months of thrice weekly aerobic training led to improved global cognitive function, as measured by Alzheimer’s disease Assessment Scale-Cognitive subscale (ADAS-Cog), compared with a health education program. Thus, we conducted secondary analyses to investigate whether these changes in global cognitive function were associated with changes in resting state DMN connectivity. A subsample of 21 participants underwent a resting state functional magnetic resonance imaging (fMRI) scan before and after trial completion. Change in resting state DMN connectivity was found to significantly predict change in ADAS-Cog score (β = -.442, p=.038) after controlling for age, intervention group, and baseline functional capacity (R2=.467, F(4,16)= 3.507, p=.031). These findings suggest that functional connectivity of the DMN may underlie changes in global cognitive function. Furthermore, aerobic exercise is a promising intervention by which to elicit these changes in older adults with mild SIVCI.

